# Application of error-prone PCR to functionally probe the morbillivirus Haemagglutinin protein

**DOI:** 10.1099/jgv.0.001580

**Published:** 2021-04-08

**Authors:** Giulia Gallo, Carina Conceicao, Christina Tsirigoti, Brian Willett, Stephen C Graham, Dalan Bailey

**Affiliations:** ^1^​ The Pirbright Institute, Guildford, Surrey, GU24 0NF, UK; ^2^​ MRC University of Glasgow Centre for Virus Research, Glasgow, UK; ^3^​ Department of Pathology, University of Cambridge, Tennis Court Road, Cambridge CB2 1QP, UK

**Keywords:** morbillivirus, epPCR, viral evolution, directed evolution, viral entry, peste des petits ruminants virus

## Abstract

The enveloped morbilliviruses utilise conserved proteinaceous receptors to enter host cells: SLAMF1 or Nectin-4. Receptor binding is initiated by the viral attachment protein Haemagglutinin (H), with the viral Fusion protein (F) driving membrane fusion. Crystal structures of the prototypic morbillivirus measles virus H with either SLAMF1 or Nectin-4 are available and have served as the basis for improved understanding of this interaction. However, whether these interactions remain conserved throughout the morbillivirus genus requires further characterisation. Using a random mutagenesis approach, based on error-prone PCR, we targeted the putative receptor binding site for SLAMF1 interaction on peste des petits ruminants virus (PPRV) H, identifying mutations that inhibited virus-induced cell-cell fusion. These data, combined with structural modelling of the PPRV H and ovine SLAMF1 interaction, indicate this region is functionally conserved across all morbilliviruses. Error-prone PCR provides a powerful tool for functionally characterising functional domains within viral proteins.

The morbilliviruses are a conserved group of pathogens that infect mammals. The prototypic virus measles virus (MeV) has a well-established human tropism, while other viruses infect small ruminants (peste des petits ruminants, PPRV), carnivores (canine distemper virus, CDV), aquatic mammals (porcine distemper virus, PDV, and cetacean morbillivirus, CeMV) and cattle (the now eradicated rinderpest virus, RPV). A characteristic feature of these viruses is their conserved usage of proteinaceous receptors, SLAMF1 or Nectin-4, to enter cells, respectively. SLAMF1, identified in 2000 [1] is expressed on immune cells, including specific subsets of T and B cells as well as macrophages and dendritic cells, with infection contributing to leucopoenia and immunosuppression. Nectin-4, independently identified by two laboratories in 2011 [2, 3], is a component of the adherens junction on polarised epithelial cells, and expressed in the nasopharynx, trachea and various other tissues [4]. The dual-tropism of morbilliviruses for these specific receptors is now understood to contribute to their significant pathogenicity and efficient onward transmission [2] and the mechanistic processes underpinning the entry of morbilliviruses is now relatively well characterised. In a multi-step process the tetrameric viral Haemagglutinin protein (H), a type II membrane glycoprotein, embedded in the viral membrane attaches to these cellular receptors via the globular head of its ectodomain, found at the C terminus of the polypeptide [5]. Such binding triggers conformational changes in these head domains, which through a process involving the four-helix bundle stalk (N-terminal; amino acids 59–154) of H, ultimately activates the trimeric, proteolytically pre-processed, viral Fusion protein (F), a type I membrane glycoprotein, leading to unfolding of this oligomer and insertion of the fusion peptide in the plasma membrane [6]. Similar processes occur during both viral-cell fusion and cell-cell fusion, the major routes of morbillivirus entry into cells. Recent work by our lab and others in the field has highlighted how the specificity of this receptor usage may influence the host-range of individual viruses. This has been led by experimental identification of mutations that confer additional receptor tropisms (D540G in CDV H [7]) as well as structure-guided approaches to identify similar restrictions (R191P in PPRV H [8]), mutations which are both within the receptor binding domain (RBD) of morbillivirus H proteins. However, many of the structural conclusions drawn from this work are based on assumptions that the RBD of all morbillivirus H proteins is structurally similar to the complex solved for MeV H and marmoset SLAM (maSLAM) [9], despite evidence that there may be subtle differences in the interaction site [10]. Our initial aim with this study was to use an unbiased mutagenic screening approach to confirm the importance of this proposed RBD of PPRV H for binding to the receptor SLAMF1.

More generally, however, we were also interested in developing tools that provide alternatives to classical gain of function experiments, i.e. the forced adaptation of morbilliviruses to non-cognate receptors followed by genome sequencing to identify mutations associated with phenotypic changes in receptor usage, an approach safely and successfully applied by other laboratories [7]. Previously, we have used a process of informed mutagenesis, based on structure-guided modelling to predict the role of specific amino acids in host-receptor tropism [8]. However, this approach has its limitations and does not recapitulate the random mutagenesis and natural selection process that RNA viruses use to rapidly evolve when under bottleneck selection. To this end we devised a protocol to randomly mutagenize the proposed RBD.

The structure of MeV H bound to SLAMF1 was solved in 2011, identifying four key motifs within a large receptor binding domain (RBD) in the globular head with a contact area of 1050 Å^2^ [9]. The majority of the RBD for SLAMF1 lies within the C terminus of the H polypeptide, corresponding to β-propellers 5 and 6, and is composed of hydrophobic interactions and salt bridges (sites 1, 2 and 4) as well as intermolecular β-sheet interactions (site 3) between H and SLAMF1. Although site 3 is in close proximity to sites 1, 2 and 4 (aa 505–554), this β-sheet (aa 191–195) is distal on the polypeptide, closer to the stalk and its linker domain [5, 9, 11]. This RBD overlaps to some degree with the site of Nectin-4 interaction which lies in a groove between propellers 4 and 5 [6, 12]. Interestingly, receptor binding on the side of the morbillivirus H β-barrel contrasts with related paramyxoviruses such as human parainfluenza 3 (HPIV3; a respirovirus) and Hendra virus (HeV; a henipavirus) where receptor recognition is localised to the central cavity (reviewed in detail in Navaratnarajah *et al*. [6]). Our own work mutating key residues in the RBD (specifically position 191 within the intermolecular β-sheet interaction of site 3 [8, 9]), together with modelling of this interaction, indicates that the PPRV and ovine (sheep) SLAMF1 RBD is likely to be structurally similar, and important in determining host-range ([8], Fig. 1a). To functionally screen the importance of this region in PPRV we adopted a random mutagenesis approach driven by error prone PCR (epPCR) (described in detail in [13]), targeting a PPRV H cloned from a field isolate of this virus (AJ849636 [14]). Using this approach, a library of increasingly mutagenized PCR products was generated, analogous to the process of forcibly driving RNA virus populations towards error catastrophe with chemical mutagens such as favipiravir (Fig. 1b) [15]. To focus our mutagenesis on the PPRV H RBD we combined this approach with an overlap PCR amplification protocol to generate a library of PCR products with a ‘faithful’ N terminus and a mutagenized C terminus (Fig. 1c). Of note, this approach did not therefore target site 3 of the RBD, or the stalk and its linker, for mutagenesis since they are, as discussed earlier, distal on the polypeptide. In total 64 cycles of epPCR were performed with nascent product moved to a new tube every four cycles (as described in [13]), yielding 16 products with an increasingly complex mix of mutagenized DNA (Fig. S1a, available in the online version of this article). To minimise downstream handling the epPCR products were then pooled into four libraries (1–4, 5–8, 9–12 and 13–16), with the range representing the epPCR products, before being used as templates for generation of full-length DNA products by standard PCR (L1-4, L5-8, L9-12 and L13-16; Fig. S1b). A ‘faithful’ wild-type H sequence (WT) was also amplified as a positive control. All products were amplified with primers containing an N-terminal T7 promoter sequence and restriction enzyme sites for cloning. To validate our hypothesis that random mutagenesis of PPRV H would lead to a loss of binding to ovine SLAMF1 we examined the capacity of these libraries to drive cell-cell fusion in transfected cells (described in Fig. 1d). HEK293T effector cells, initially infected with a recombinant fowlpox virus expressing T7 polymerase, were transfected with H-expressing PCR products, as well as plasmids expressing PPRV F and one-half of a split GFP-Luciferase reporter (rLuc-GFP 1–7 [16]). Separately, HEK293T target cells were transfected with plasmids expressing ovine SLAMF1 and the other half of the reporter (rLuc-GFP 8–11). Whereas the WT PCR product generated a robust level of cell-cell fusion, the epPCR libraries, as expected, showed decreasing levels of fusion as the epPCR cycle number increased. Indeed, effector cells expressing library L13-16 did not show levels of fusion above those seen with the negative control target cells bearing no receptor (Fig. 1e).

**Fig. 1.**
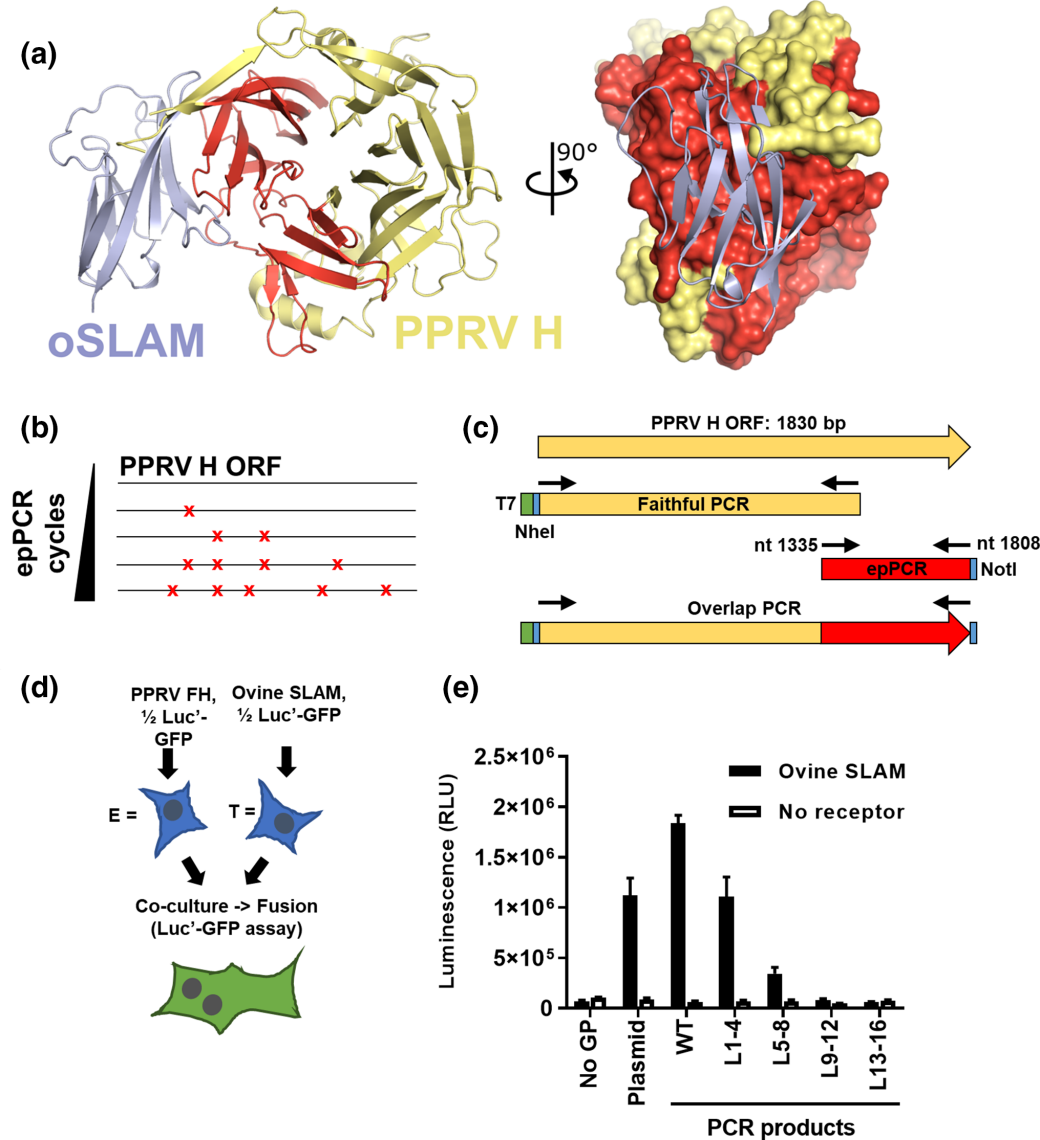
Error-prone PCR mutagenesis of PPRV H. (**a**) A model of ovine SLAM (oSLAM, blue ribbons) in complex with PPRV H (yellow and red ribbons), generated using the structure of MeV H in complex with marmoset SLAM (PDB ID 3ALX [9]) is shown in two orthogonal views. In the right-hand view the molecular surface of PPRV H is shown. Residues that were mutagenized by epPCR are coloured red. Model available at https://doi.org/10.17863/CAM.58532. (**b**) A simplified schematic of epPCR illustrating the correlation between increasing cycle number and the number of mutations (red crosses). (**c**) epPCR libraries of the PPRV H ORF were generated using Taq polymerase (Promega), as described in [13] with a 3′ primer including a NotI restriction site, and 64 cycles yielded 16 PCR products in total (represented by red bar; see Fig. S1a). These were subsequently pooled into four libraries (1–4, 5–8, 9–12 and 13–16; see Fig. S1b). Seperately, a faithful PCR copy of the N terminus (yellow) was amplified by standard high-fidelity PCR (using Kod polymerase, Merck) with a 5′ primer containing a T7 promoter sequence (green) and the NheI restriction site (blue). Lastly, overlap PCR was performed using the two external primers and Kod high-fidelity PCR to generate four libraries containing PCR products with full-length PPRV ORFs (yellow/red, L1-4, L5-8, L9-12 and L13-16 see Fig. S1b). (**d**) The quantifiable cell-cell fusion for PPRV relies on co-transfection of effector cells [E] with constructs expressing PPRV F and H proteins as well as half of a rLuc-GFP dual reporter [16]. These are co-cultured with target cells [T] expressing ovine SLAM and the corresponding half of the reporter. A cognate combination of FH and receptor leads to cell-cell fusion, reconstitution of the reporter and activation of the luciferase and GFP proteins. (**e**) Increasing epPCR mutagenesis of the PPRV H protein leads to reduced cell-cell fusion. Effector cells were transfected with the split reporter and no glycoprotein (No GP), plasmids bearing WT PPRV F and H (Plasmid), a plasmid expressing F and a PCR product expressing the PPRV H ORF (WT) amplified using the same external primers used in (**c**), or, the overlapped PCR products from this same epPCR protocol. After 2 days these were co-cultured with target cells expressing the corresponding half of the split reporter and either mock vector (No receptor) or a plasmid expressing ovine SLAMF1 (Ovine SLAM). Luciferase activity was measured using a cell permeable Renilla luciferase substrate (Coelenterazine) the following day. Assays were performed in biological triplicate with the mean and standard deviation plotted.

To identify and characterise individual mutants we adopted a stepwise screening procedure involving the cloning of the epPCR PPRV H libraries into an expression vector (pcDNA3.1) and the subsequent examination of cell-cell fusion in a plasmid-transfection based system that does not require recombinant fowlpox virus [8]. To facilitate screening, we initially generated pools containing 16 individual colonies, with three pools per library (denoted A to C for each of the four epPCR libraries, Fig. 2a). Screening of the pools (L1-4A through L13-16C, where each contains 16 constructs) indicated that the first library (pools L1-4A, L1-4B and L1-4C) had maintained approximately 50 % fusion activity (Fig. 2b). To avoid the characterisation of non-mutated (wild-type, WT) clones in library L1-4 we focused our screening on the subsequent libraries, where evidence of fusion was more sporadic, our goal being the identification of a mixture of functional and non-functional H proteins. Choosing the pools L5-8A, L5-8C and L13-16B, where fusion activity was present (and discarding the rest where little evidence of fusion remained) we sub-divided each of these into four sub-pools for further characterisation (each containing four constructs), subsequently identifying four with functional phenotypes (Fig. 2c; L5-8A/1, L5-8C/2, L5-8C/3 and L13-16B/4). At this final stage we then characterised and sequenced all individual mutants in these sub-pools (16 constructs sequenced in total), identifying four that had retained robust activity in the cell-cell fusion assay (L5-8A/1A, L5-8C/2D, L5-8C/3D and L13-16B/4A) (Fig. 2d, e; green text). Of note, at each step the pools (and finally individual clones) were analysed by restriction digest to confirm the insertion of the H ORF (Fig. S1c–e).

**Fig. 2.**
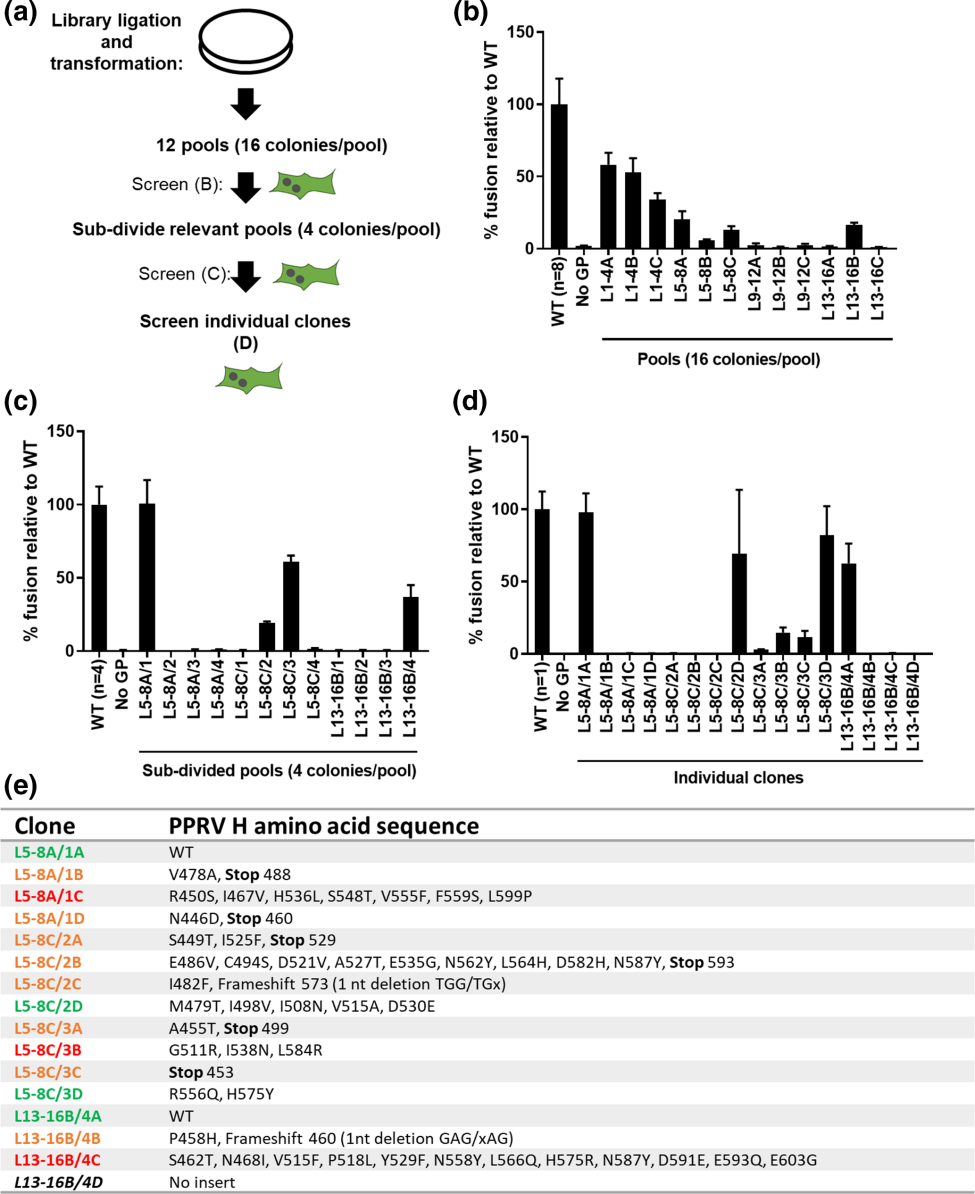
Screening of PPRV H epPCR libraries to identify individual mutants and link phenotype to genotype. (**a**) The pool selection and deconvolution strategy for identifying individual H mutants with modified cell-cell fusion activity (see main text for detailed explanation). (**b**) Screening of the pools L1-4A to L13-16-C (each containing 16 colonies) using a PPRV cell-cell fusion assay, as described in Fig. 1, identified a correlation between increased epPCR cycle number and decreased cell-cell fusion activity (similar to Fig. 1e). For WT a selection of colonies expressing the unmutated PPRV H was used (*n*=8 or as shown). (**c**) Pools L5-8A, L5-8C and L13-16B were taken forward for further analysis by sub-dividing the pools into four colonies per pool (e.g. L5-8A/1 to L5-8A/4) and re-examining their cell-cell fusion phenotype. (**d, e**) Based on these results pools L5-8A/1, L5-8C/2, L5-8C/3 and L13-16B/4 were separated into their individual colonies, re-screened in the cell-cell fusion assay and each mutant’s PPRV H ORF sequenced between nt 1335 and 1808 – the region targeted by epPCR – with any mutations to the PPRV H amino acid sequence calculated. Clone numbers in (**e**) are coloured based on mutation type and relative activity in the cell-cell assay (green=high activity relative to WT, orange=frameshift or premature stop codon, red=little or no activity relative to WT). Luciferase activity was measured using a cell permeable Renilla luciferase substrate (Coelenterazine). Assays were performed in biological triplicate with the mean and standard deviation plotted. Experiments in panel D were performed three separate times with a representative data set shown.

The four functional mutants had a combination of genotypes including two with WT amino acid (aa) sequences, a single mutant with the mutations M479T/I498V/I508N/V515A/D530E and another with R556Q/H575Y. That L5-8A/1A expressed a WT PPRV H aa sequence was perhaps not surprising, given the relatively low number of associated epPCR amplification cycles (20-32); however, the presence of a similar genotype in L13-16B/4A was more interesting. In this case the related cycle number is much higher (52-64) indicating that, even at the final stages of the epPCR process, WT PCR products exist within the population. Nevertheless, by identifying mutant genotypes that sustained fusion the epPCR process had identified functional plasticity within PPRV H at specific aa residues. More detailed examination of these residues, informed by the modelled structure of PPRV H with oSLAM, provides some context for this maintenance of function (Fig. 3a). The I498V mutation in L5-8C/2D, for example, is a surface exposed isoleucine which is close to the predicted oSLAM RBD; however, the conservative substitution to valine is unlikely to change the hydrophobicity of this region. Likewise, the V515A and D530E mutations, although buried in the protein interior, are conservative mutations unlikely to dramatically alter protein structure or function. In L5-8C/3D the H575Y mutation reflects changes to a surface exposed residue distant from the RBD, with substitution to tyrosine likely to be well tolerated. Supporting this conclusion, amino acid alignments of MeV, RPV, CDV and PPRV H proteins show that this residue is highly variable with glutamine, arginine and aspartic acid being found in MeV, RPV and CDV strains, respectively (data not shown). The R556Q mutation is interesting as this residue is more conserved in morbilliviruses (arginine in PPRV, MeV and CDV, and lysine in RPV) and closer to the RBD, specifically sites F552 and P554 – components of sites 2 and 4 in the MeV H RBD characterised by Hashiguchi *et al*. [9]. However, this residue does not form well-ordered salt bridges or hydrogen bonds in the MeV-maSLAM model [9]; perhaps explaining why the glutamine side chain is well-tolerated from a functional perspective (Fig. 3a).

**Fig. 3.**
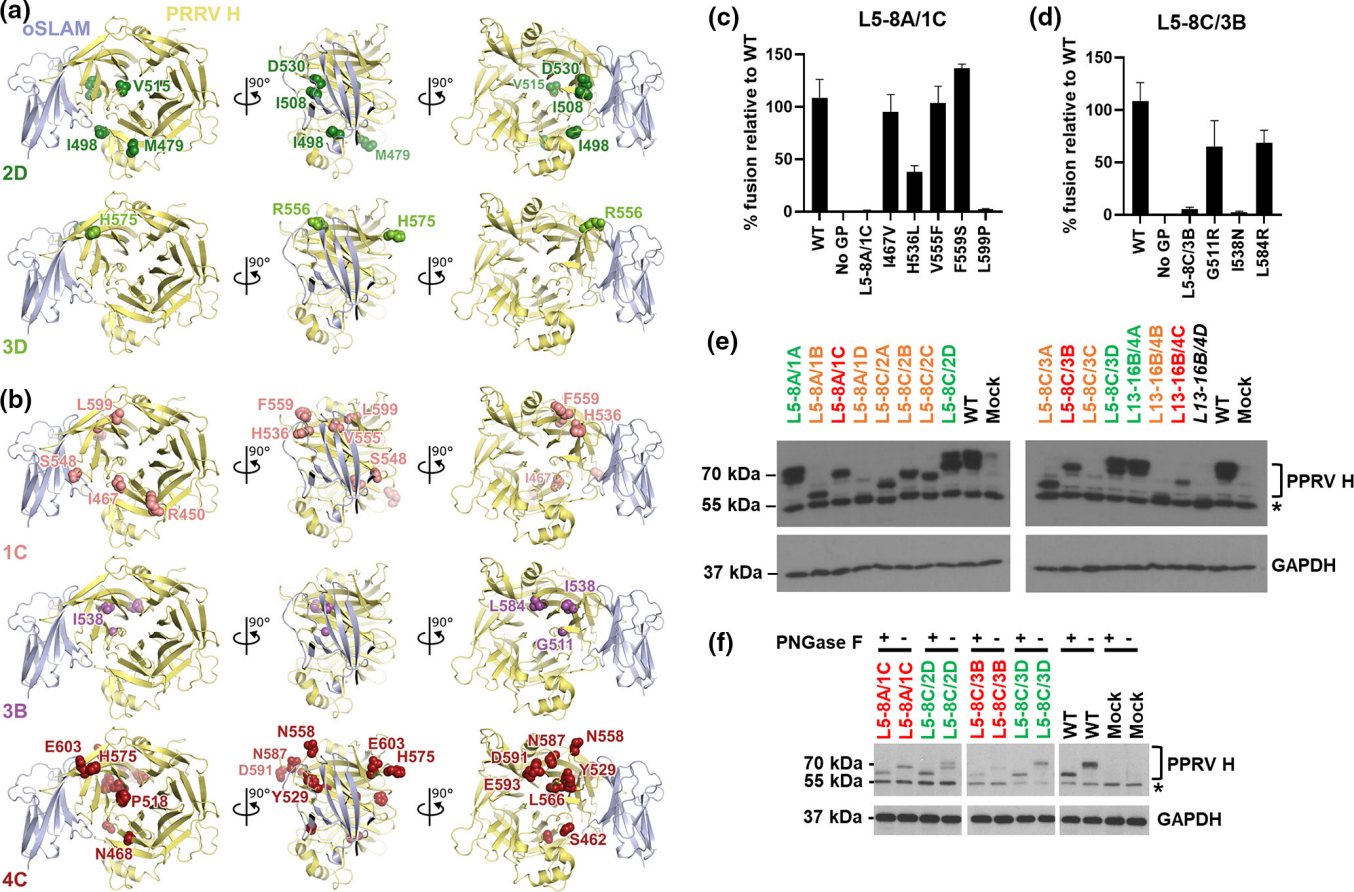
Examining the predicted structural configuration, electrophoretic mobility and glycosylation pattern of individual PPRV H mutants. (**a, b**) A model of ovine SLAM (oSLAM, blue ribbons) in complex with PPRV H (yellow ribbons) is shown in three orthogonal views. (**a**) Residues that could be mutated without abolishing PPRV H binding to oSLAM are shown in spacefill, coloured light green (L5-8C/3D) or dark green (L5-8C/2D). (**b**) Residues that prevented SLAM binding when mutated are shown in spacefill, coloured pink (L5-8A/1C), magenta (L5-8C/3B) or red (L13-16B/4C). (c, d) Generation of individual point mutations present in L5-8A/1C and L5-8C/3B, and their characterisation in a PPRV cell-cell fusion assay, identified mutations H536L, L599P and I538N as being particularly deleterious to receptor usage. Assays were performed in biological quadruplicate with the mean and standard deviation plotted. Experiments were performed three times with a representative data set shown. (**e**) HEK293T cells were transfected with plasmids expressing the PPRV mutant (coloured as indicated in Fig. 2e) and lysates harvested at 24 h for Western blot with an antibody detecting the cytoplasmic tail (not targeted by epPCR) of PPRV H. GAPDH Westerns were included as protein loading controls. (**f**) The glycosylation of individual PPRV H mutants, or the WT H, was examined using Peptide: N-Glycosidase F (PNGaseF; New England Biolabs) as per the manufacturer’s instructions. Treated or untreated lysates were prepared and examined as per (**c**). * The band at 55 KDa in the PPRV H Western is a non-specific cellular protein.

Another interesting consequence of the epPCR process was the introduction of a high proportion of stop codons, with six clones (38%) presumably losing cell-cell fusion functionality in this way (Fig. 2e; orange text). Of note, one of these mutants, L5-8C/3C repeatedly showed activity in our assay (Fig. 2d); however, the subsequent re-isolation of six individual clones of this construct did not, indicating this activity was likely the result of contamination, possibly WT plasmid (data not shown). Elsewhere, two frameshifts were observed, the result of 1 nt deletions, both guanidines, highlighting the potential for this protocol to erroneously remove bases from the nascent strand (Fig. 2e; orange text). A final non-functional mutant lacking the epPCR-generated region of H was also identified (L13-16B/4D); however, this is likely an artefact of cloning. In contrast, a number of full-length ORF-containing clones with little or no functionality in the cell-cell fusion assay were also identified; L5-8A/1C (R450S/I467V/H536L/S548T/V555F/F559S/L599P), L5-8C/3B (G511R/I538N/L584R), and L13-16B/4C (S462T/N468I/V515F/P518L/Y529F/N558Y/L566Q/H575R/N587Y/D591E/E593Q/E603G), respectively (Fig. 2d, e; red text), identifying aa sites in PPRV H where mutations are poorly tolerated. Examination of the location of these residues on our modelled interaction of PPRV H and oSLAM identified several substitutions likely to be responsible for the observed loss of function (Fig. 3b). Within L5-8A/1C, the H536L substitution would prevent the formation of a side-chain H-bond with Y529, which may cause local structural rearrangements in the region close to the oSLAM binding site. In addition, substitution of the buried L599 residue with proline would probably cause significant structural rearrangement of the H β-propeller number 5, since the constrained backbone conformation of proline (ϕ=−60°±20°) is not compatible with the phi angle adopted by L599 (ϕ=−131.6°). The specific deleterious role of H536L and L599P in the loss of function of clone L5-8A/1C, in contrast to other changes including I467V, V555F and F559S, was confirmed by individual point mutation of these residues in a WT PPRV H clone (Fig. 3c). With reference to L5-8C/3B the I538N mutation is close to predicted interaction motifs within the RBD [9] and introduction of a polar residue into this hydrophobic cavity is likely to be poorly tolerated, causing significant structural rearrangement. Again, the individual importance of this mutation was subsequently confirmed by standard targeted mutagenesis of the WT PPRV H clone (Fig. 3d). Lastly, within L13-16B/4C four mutations (N468I, V515F, Y529F and L566Q) stand out as being particularly deleterious. N468 is a buried polar residue in proximity to RBD motifs thought to interact with oSLAM. Introduction of a non-polar residue at this site would prevent formation of hydrogen bonds with Y410, destabilising the local structure. Unlike the conservative V515A mutation found in the functional mutant L5-8C/2D, the introduction of a large hydrophobic phenylalanine (V515F) is likely to cause disruption to the core folding of PPRV H. Furthermore, the Y529F change, leading to the loss of the terminal hydroxyl group of this side-chain, would prevent H-bonds forming with the O(carbonyl) of G561 and the side chain of H536, causing local disruption and likely affecting oSLAM binding. Finally, the L566Q mutation, leading to the substitution of a buried hydrophobic residue with glutamine, a polar amino acid, would again cause local disruption, although this is further from the SLAM interface and it is unclear how this might affect binding. In conclusion the mutants L5-8A/1C, L5-8C/3B and L13-16B/4C are all predicted to have deleterious mutations leading to significant structural rearrangement of H and/or the oSLAM RBD, fitting with their considerable loss of function in cell-cell fusion assays.

Western blot of the proteins produced from each of these clones, using an antibody targeting the cytoplasmic tail of PPRV H (an epitope found at the N-terminus of the polypeptide and therefore not sensitive to mutation by epPCR), was also performed. This correlated well with the sequence information and identified a number of interesting features of the H protein. Firstly, the introduction of premature stop codons or frameshifts (creating various C-terminal truncations) does not dramatically alter the relative stability of many of the polypeptides, with some being produced to near WT levels (Fig. 3e; orange text). Secondly, all the functional full-length mutants were expressed as two distinct bands on the Western blot with electrophoretic mobility corresponding to masses of ~70 kDa (Fig. 3e – green text), while the non-functional mutants either did not have the higher band (L5-8A/1C and L5-8C/3B) or were poorly expressed (L13-16B/4C). One possible explanation for the lack of the higher molecular weight band in L5-8A/1C and L5-8C/3B is the loss of specific post-translational modifications to PPRV H. To examine this in more detail we performed PNGaseF treatment of these mutant Hs (L5-8A/1C and L5-8C/3B), comparing them to L5-8C/2D, L5-8C/3D and WT H. PNGaseF is an amidase, which cleaves between the innermost GlcNAc and asparagine residues of high mannose, hybrid, and complex oligosaccharides and can remove almost all N-linked oligosaccharides from glycoproteins. Treatment with this enzyme reduced all H proteins to a uniform lower molecular weight, confirming glycosylation as the main determinant in the varying electrophoretic mobility of the H proteins (Fig. 3f). However, in the untreated controls the two functional full length mutants (L5-8C/2D and L5-8C/3D) again migrated at a higher molecular weight, with the two non-functional mutants (L5-8A/1C and L5-8C/3B) running at intermediate positions. We postulate that the upper band represents H proteins with more mature glycans, while the intermediate band may contain immature glycans, a consequence of protein misfolding. For other mutants, for example L13-16B/4C, the large number of structurally deleterious mutations (in particular N468I, V515F, Y529F and L566Q; discussed above) is likely to have affected overall protein stability, leading to degradation and the lower signal seen by immunoblotting.

Using a combination of epPCR and a relatively high throughput screening tool we were able to functionally characterise domains within the PPRV H protein proposed to contain the RBD. This included random mutagenesis of important residues for SLAMF1 interaction, e.g. D530E in L5-8C/2D, which although absolutely conserved in morbilliviruses appeared well tolerated, as well as those in close proximity, e.g. V555F in L5-8A/1C and R556Q in L5-8C/3D. That PPRV H could tolerate the V555F change is perhaps not surprising as a phenylalanine at this position is actually more common in morbillivirus H proteins. In general, the identification of a number of non-functional yet full-length mutants supports our assumption that there is structural analogy between PPRV and MeV H, not surprising given the aa similarity of the two proteins (67 % conserved). A low number of conservative changes within the epPCR targeted region were reasonably well tolerated; however, as more significant structural changes were introduced, and the number of mutations increased, the functionality of the encoded proteins decreased. Inspection of our structural model suggests that the majority of deleterious mutations prevent SLAM binding by disrupting the fold of PPRV H, e.g. H536L and L599P in L5-8A/1C (Fig. 3b, c) as well as H536L in L5-8A/1C (Fig. 3b, d) consistent with predictions that most disease-causing human SNPs affect protein stability [17]. Whilst our approach did not probe the role of the stalk domain of H (residues 59–154) it is worth noting that, post-receptor engagement, this domain is also important in triggering fusion, through its interactions with F. Although this region has been characterised in more detail for MeV [11, 18] a detailed mutational analysis of the stalk of PPRV H is also warranted.

Broader evaluation of the strengths of this epPCR approach can also be made. This technique is clearly useful when an associated 3D structure is available. Indeed, in our experiments cross-referencing structural models with the results of our epPCR mutagenesis allowed detailed functional hypotheses to be developed and tested. However, epPCR generated more misfolding mutations, than mutations at the receptor-binding surface, in PPRV H. This, combined with the high frequency of stop codons inserted, might suggest site-directed mutagenesis, where more targeted mutations to the RBD is achievable, remains the preferred approach as well as more in-depth mutational scanning approaches recently established for SARS-CoV-2 spike protein [19]. The technique is also dependent on a tractable functional phenotypic screen, although this is true of all directed evolution approaches where random mutagenesis is applied to simulate natural rates of error. Nevertheless, epPCR could represent a very powerful starting point for viral proteins which are known to be intrinsically unstructured, or where the interacting region has been mapped to a disordered region of the structure. In this case, issues with misfolding proteins are less of a concern, and epPCR might be useful for identifying binding domains or other motifs of interest within these viral proteins.

From an RNA virus evolution perspective, whilst our epPCR library generation effectively mimics the mutations caused by an error prone viral RNA polymerase, and we can apply a bottleneck (in this case loss of functionality in a cell-cell fusion assay), we could not take advantage of ‘natural selection’ and ‘survival of the fittest’ since our mutants were plasmid- rather than virally-encoded. We did attempt to clone the H mutants into a lentiviral expression system to rescue libraries of H-encoding PPRV pseudotypes; however, the efficiency of morbillivirus pseudotyping was too low (data not shown). Another caveat of this approach is that a very low mass of DNA is needed to avoid the generation of mosaic pseudotypes, concerns that have been addressed elsewhere with more efficient packaging systems [20, 21].

To summarise, the application of epPCR to functionally characterise a proposed functional domain within a viral protein, in this case PPRV H, provided a valuable set of mutations for downstream analysis. Combining this tool with a high throughput functional assay provides an effective mechanism for rapid characterisation of viral proteins.

## Supplementary Data

Supplementary material 1
