# Supplementary material for: Application of error-prone PCR to functionally probe the morbillivirus Haemagglutinin protein
Source: J Gen Virol. 2021 Apr 8;102(4):001580. doi: 10.1099/jgv.0.001580 (PMC8290269; doi:10.1099/jgv.0.001580)
Supplement: Supplementary material 1 [file jgv-102-1580-s001.pdf]

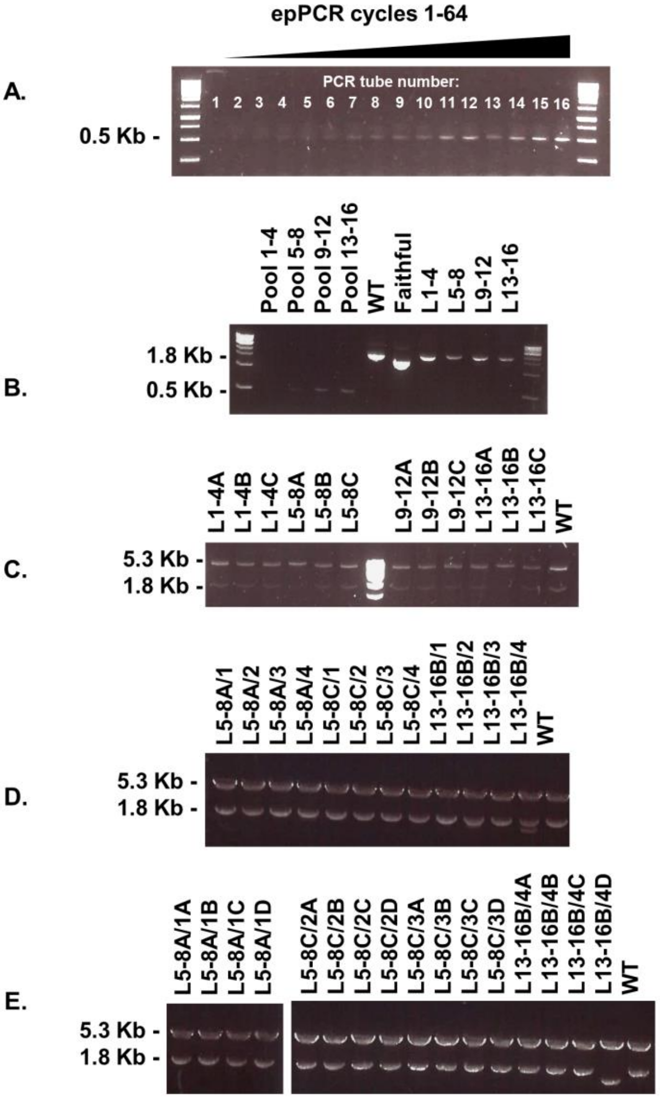

Supplementary Figure 1: epPCR of PPRV H and restriction endonuclease screening of pools and individual clones. (A) 5 µl of each epPCR product (from tubes 1-16, representing 64 total cycles [4/tube]) from the protocol described in Fig.1C was analysed on a DNA agarose gel. Expected size of epPCR band was c. 500bp. **(B)** DNA from tubes 1-4, 5-8, 9-12 and 13-16 was pooled and together with a faithful N-terminus of PPRV H (Faithful) overlapped to make epPCR libraries L1-4, L5-8, L9-12 and L13-16. A WT product representing an unmutated full-length PPRV H ORF was also generated using the same external primers, which contained a 5' NheI site and T7 promoter and a 3' NotI site. **(C-E)** Pools and individual clones from the cloned libraries were examined by restriction mapping (NheI and NotI). All gels were 1% TBE DNA agarose gels run together with a 1Kb DNA ladder (shown for A, B and C only).
